# Prevalence of migraine and tension‐type headache among undergraduate medical students of Kathmandu Valley: A cross‐sectional study

**DOI:** 10.1002/hsr2.747

**Published:** 2022-08-08

**Authors:** Oshan Shrestha, Sagun Karki, Niranjan Thapa, Kabindra Lal Shrestha, Aayushama Shah, Pramita Dhakal, Prashant Pant, Sunil Dhungel, Dhan Bahadur Shrestha

**Affiliations:** ^1^ College of Medicine Nepalese Army Institute of Health Sciences Kathmandu Nepal; ^2^ Department of Critical Care Karnali Academy of Health Sciences Jumla Nepal; ^3^ Department of Physiology Nepalese Army Institute of Health Sciences Kathmandu Nepal; ^4^ Department of Internal Medicine Mount Sinai Hospital Chicago Illinois USA

**Keywords:** headache, medical students, migraine, prevalence, primary, tension‐type

## Abstract

**Background:**

Headache is the most prevalent neurological symptom which can be of a serious condition, as in brain tumor, but mostly it is a benign condition that includes primary headache such as migraine or tension‐type headache (TTH). Migraine reoccurs frequently and is more severe but owing to the high prevalence of TTH, however, impaired quality of life due to TTH is greater than that of migraine at the population level. Medical students are constantly subjected to stress and in such a condition, it was necessary to find out the burden of headache among medical students. This cross‐sectional study done among medical students aims to generate some data and literature which will change the outlook of stakeholders towards headache disorders among medical students.

**Methods:**

This cross‐sectional study is based upon Headache Screening Questionnaire—English Version questionnaire based upon the ICHD‐3 beta criteria. Medical students of Kathmandu valley were sampled by using convenient sampling and data were collected. Data were refined in Microsoft Excel and imported to SPSS 20 for analysis.

**Results:**

A total of 352 individuals were part of this study, out of which 229 (65.1%) were males and 123 (34.9%) were females with a mean age of 21.72 ±  1.601 years (mean ± SD). Prevalence of migraine and TTH was found to be 15.3% (95% confidence interval [CI]: 11.7%–19.3%) and 40.3% (95% CI: 34.9–45.2), respectively. Through multivariate binomial regression, it was observed that the odds of being diagnosed with migraine increased with age (adjusted odds ratio [AOR] = 1.266 [1.013–1.583], *p* = 0.038), females had twice the odds of experiencing migraine headaches compared to males (AOR = 2.119 [1.074–4.180], *p* = 0.03), and medical students who stayed at the hostel were at lesser odds of experiencing migraine headache (AOR = 2.772 [1.501–5.118], *p* = 0.01).

**Conclusion:**

Prevalence of migraine and TTH among undergraduate medical students was found to be 15.3% and 40.3%, respectively.

## 1 BACKGROUND

Headache is the most prevalent neurological symptom which is experienced by almost everyone at least once in their lifetime.^1^ This neurological symptom can be of a serious condition, as in brain tumor, but mostly it is a benign condition that includes primary headache such as migraine or tension‐type headache (TTH).^2^ Migraine and TTH are a matter of importance to global public health because they impose a widespread burden of ill health and impaired quality of life.^3^

International Headache Society differentiates migraine and TTH according to their frequency of occurrence, severity, intensity, anatomical involvement of the head, aggravating factors, and associated factors. In contrast to TTH, migraine reoccurs frequently, is more severe, has unilateral involvement, pulsating quality, is aggravated by routine physical activities, and is associated with nausea and/or photophobia and phonophobia.^4^ But, owing to the high prevalence of TTH (globally, 11% for migraine, 42% for TTH), impaired quality of life due to TTH is greater than that of migraine at the population level.^3^ The burden of ill health and impaired quality of life due to headache remains large and it is estimated that it costs a minimum of US$100 million per million inhabitants per year.^2^ In a domestic study within Nepal, headache was seen as one of the most prevalent painful conditions.^5^

Medical students are constantly subjected to stress regarding their academics, performance, exams, and responsibilities. In such circumstances, medical students are prone to experience headaches and this study aims to find out the prevalence of primary headache (migraine and TTH) among undergraduate medical students. This study also aims to act as a nidus for future large‐scale studies among medical students for necessary intervention. This study is in line with STROBE guidelines.^6^

## 2 METHODS

This cross‐sectional study based on a self‐administered questionnaire was carried out among the undergraduate medical students of Kathmandu Valley from October 22, 2021 to February 20, 2022. List of students from a total of six medical colleges along with their contact information was accessed. Selected ones were sent the weblink to Google Forms containing the questionnaire through different internet platforms. Before the questionnaire page opened, each respondent was asked for their consent compulsorily and only those who gave the consent were allowed to go to the next page. Ethical clearance for this study was taken from the Nepalese Army Institute of Health Sciences Institutional Review Committee (Ref no: 455). All of our respondents were informed about the nature of the study by including the written consent form in the questionnaire itself. All the participants were assured of confidentiality.

### 2.1 Sample size

For sample size calculation we used Cochran's formula considering the heterogenous and large population.^7^ Details of sample size calculation is available as Supporting Information: File S1.

$$\begin{array}{l} n=Z^{2}\times \left(p\times q\right)/e^{2}=1.96^{2}\times \left(0.5\times 0.5\right)/0.05^{2}=384 \end{array},$$

where *n* is calculated sample size; *Z* is 1.96 at 95% confidence interval (CI); *p* is expected prevalence of students having headache, 50%; *q* = 1 − p; *e* is the margin of error (5%).

Total number of MBBS students in Kathmandu Valley during the study period (*N*): 2624.

$$\begin{array}{lll} \text{Adjusted sample size}\left(n'\right) & = & n/\left(1+n/N\right)\\ & = & 384/\left(1+384/2624\right)=335. \end{array}$$

Considering 8% nonresponse rate, the final sample size was 361.

We used convenient sampling to select students. List of students (from the first year to final year) from each college was accessed and respondents were selected. Through this process, 361 individuals were selected.

### 2.2 Study tool

Headache Screening Questionnaire—English Version (HSQ‐EV), based on ICHD‐3 beta, is a 10‐item questionnaire that is a sensitive screening tool. This 10‐item questionnaire is used to screen for both migraine and TTHs. A particular score has been assigned to each answer of a question and according to the answers given by the responders total score is calculated. The cut‐off point for definite diagnosis is eight and for probable diagnosis is six. When all the criteria based on ICHD‐3 beta were met, the score received would be eight for both migraine and TTH and when the score received is at least six points, migraine and TTH are considered to be probable migraine and probable TTH.^8^ Along with the HSQ‐EV, questions of sociodemographic were also included in the questionnaire. The study tool is available as Supporting Information: File S1.

### 2.3 Dependent and independent variables

All of the dependent variables (DVs) and independent variables (IVs) were dichotomous and categorical except for age. DVs were diagnosis of migraine and diagnosis of TTH. IVs included age, sex, year of study, involvement in extracurricular activities, daily exercise/yoga/outdoor sports, having a demanding family responsibility, food preference, and smoking habit. These IVs were selected after literature review.

### 2.4 Analytical strategy

Frequency was calculated for all the IVs and lifetime prevalence was calculated for DVs with CI. Binomial logistic regression was used to see how DVs and IVs affected each other, univariate analysis gave crude odds ratio and multivariate analysis gave adjusted odds ratio (AOR; no migraine/TTH was coded as 0 and presence of migraine/TTH was coded as 1. Data were refined in excel then imported to SPSS 20 for the analysis. Diagnosis of definite and probable migraine was combined in the estimation of prevalence and other analyses. The same was done for TTH.

## 3 RESULTS

Out of 361 students, a total of 352 individuals who responded were part of this study. Out of the included individuals 229 (65.1%) were males and 123 (34.9%) were females. Age of the participants ranged from 17 to 28 with a mean age of 21.72 ± 1.601 years (mean ± SD). The rest of the sociodemographic details are listed in Table 1.

**Table 1:** Characteristics of the included sample

Characteristics	Sample group (*N* = 352)	
	*n*	%
Age (in years)		
Late teen (17–19)	25	7.10
Early 20s (20–23)	280	79.54
Mid 20s (24–26)	45	12.78
Late 20s (27–28)	2	0.56
Sex		
Male	229	65.1
Female	123	34.9
Year of study		
Preclinical	144	40.9
Clinical	208	59.1
Stays at hostel		
Yes	232	65.9
No	120	34.1
Involvement in extracurricular activities		
Yes	219	62.2
No	133	37.8
Daily exercise/yoga/outdoor sports		
Yes	173	49.1
No	179	50.9
Have a demanding family responsibility		
Yes	116	33.0
No	236	67.0
Food preference		
Vegetarian	58	16.5
Nonvegetarian	294	83.5
Smoking habit		
Yes	31	8.8
No	321	91.2
Seeks medical attention for headache		
Yes	69	19.6
No	283	80.4

### 3.1 Prevalence of migraine and TTH

The prevalence of migraine was found to be 15.3% (CI: 11.7–19.3). A total of 25 out of 123 females and 29 out of 229 males had migraine. Similarly, 27 out of 144 preclinical students and 27 out of 208 clinical students were seen to have migraine headache.

The prevalence of TTH was found to be 40.3% (CI: 32.9–45.2). A total of 52 out of 123 females and 90 out of 229 males were screened to have TTH. A total of 58 out of 144 preclinical students and 84 out of 208 clinical students were found to have TTH (Table 2).

**Table 2:** Prevalence of migraine and tension‐type headache

Type of headache	Sample group (*N* = 352)			
	*n*	%	95% Confidence interval	
			Lower	Upper
Migraine				
Definite migraine	16	4.5	2.6	6.8
Probable migraine	38	10.8	7.7	14.2
Total	54	15.3	11.7	19.3
Tension‐type headache				
Definite tension‐type headache	24	6.8	4.3	9.7
Probable tension‐type headache	118	33.5	28.7	38.6
Total	142	40.3	34.9	45.2

### 3.2 Regression analysis result of migraine

Findings of univariate analysis showing crude odds ratio are presented in Table 3.

**Table 3:** Binomial logistic regression, univariate analysis

Variables	Coeff	*p* value	OR	95% CI	
				Lower	Upper
Age	0.061	0.506	1.063	0.888	1.273
Sex					
Male	Reference				
Female	0.565	0.059	1.759	0.978	3.164
Year of study					
Preclinical	Reference				
Clinical	−0.436	0.142	0.646	0.361	1.157
Stays at hostel					
Yes	Reference				
No	1.061	<0.01	2.889	1.600	5.217
Involved in extracurricular activities					
Yes	Reference				
No	0.055	0.856	1.057	0.583	1.916
Daily exercise/yoga/outdoor sports					
Yes	Reference				
No	0.047	0.873	1.048	0.587	1.873
Have a demanding family responsibility					
Yes	Reference				
No	0.080	0.802	1.083	0.581	2.018
Food preference					
Vegetarian	Reference				
Nonvegetarian	−0.169	0.661	0.845	0.398	1.794
Smoking habit					
Yes	Reference				
No	−0.065	0.899	0.937	0.343	2.557

Abbreviations: CI, confidence interval; coeff, coefficient beta; OR, odds ratio.

Model was then adjusted for age, sex, year of study, involved in extracurricular activities, daily exercise/yoga/outdoor sports, have a demanding family responsibility, food preference, and smoking habit to get AOR.

Model was statistically significant and it could distinguish between those with migraine and without the diagnosis of migraine (*χ* ^2^ = 21.171, *p* = 0.012).

Hosmer and Lemeshow test yielded a nonsignificant value, *χ* ^2^ = 4.623, *p* = 0.797, suggesting that the model fits well. Details of findings of multivariate analysis are shown in Table 4.

**Table 4:** Binomial logistic regression, multivariate analysis

Variables	Coeff	*p* value	AOR	95% CI	
				Lower	Upper
Constant (intercept)	−6.813	0.008	0.001		
Age	0.236	0.038	1.26	1.013	1.583
Sex					
Male	Reference				
Female	0.751	0.03	2.11	1.074	4.180
Year of study					
Preclinical	Reference				
Clinical	−0.676	0.06	0.509	0.245	1.055
Stays at hostel					
Yes	Reference				
No	1.020	0.01	2.77	1.501	5.118
Involved in extracurricular activities					
Yes	Reference				
No	−0.278	0.421	0.758	0.385	1.491
Daily exercise/yoga/outdoor sports					
Yes	Reference				
No	−0.067	0.836	0.935	0.496	1.763
Have a demanding family responsibility					
Yes	Reference				
No	0.092	0.784	1.097	0.567	2.121
Food preference					
Vegetarian	Reference				
Nonvegetarian	−0.163	0.688	0.849	0.382	1.887
Smoking habit					
Yes	Reference				
No	−0.201	0.721	0.818	0.272	2.460

Abbreviations: AOR, adjusted odds ratio; CI, confidence interval; Coeff, coefficient beta.

Age of the participants ranged from 17 to 28 and it is observed that the odds of being diagnosed with migraine increases with age (AOR = 1.26 [1.013–1.583], *p* = 0.038).

Females had twice the odds of experiencing migraine headaches compared to males (AOR = 2.11 [1.074–4.180], *p* = 0.03). Medical students who stayed at the hostel were at 2.77 times less odds of experiencing migraine headache (AOR = 2.77 [1.501–5.118], *p* = 0.01).

### 3.3 Regression analysis result of TTH

No significant difference in odds between categories of different DVs was observed when running binomial regression analysis. Results of univariate and multivariate analysis is available as Supporting Information: File S2.

## 4 DISCUSSION

In this article, we have studied the prevalence of migraine and TTH by using the HSQ‐EV questionnaire based upon the ICHD‐3 beta. Migraine, probable migraine, no migraine, TTH, probable TTH, and no TTH were the possible domains in which the respondents could be grouped through this questionnaire. Probable migraine refers to migraine‐like attacks but without one of the features required to meet all the criteria for migraine, and also not fulfilling the criteria for any other headache types.^4^ This study done among the medical students in Kathmandu is the first of its kind in the country. In our study, the prevalence of Migraine was found to be 15.3% and the prevalence of TTH was found to be 40.3%. Furthermore, among similar studies done across the world, the prevalence of migraine in Enugu, Nigeria was similar.^9^ While some studies from India,^10^ Saudi Arabia,^11^ Turkey,^12^ and Kuwait^13^ shows a higher prevalence of migraine among medical students, whereas another study from South‐East Iran^14^ shows lower prevalence. This wide range of prevalence across the world may be attributed to the geography and altitude,^15^ cultural differences, data collected at different periods of time, and different tools of measurement.

Another significant finding of this study suggested that females are at 2.11 more odds (AOR) of experiencing migraine headaches compared to males. Similar findings were observed in other studies.^11^, ^13^, ^16^ Migraine often has a close relationship with the menstrual cycle of women,^4^ however, to include the menstrual history to differentiate the classical migraine from menstrual migraine was out of the scope of this current study. The mechanism behind headache during menstruation is not entirely clear but could be traced back to estrogen deficiency^17^ and this is yet to be explored in future studies. Our study reports that nonhostellers are at 2.77 more odds (AOR) of having migraine which is in disagreement with a study by Narang and Jahan,^18^ which reported that nonhostellers have less level of stress as well as sleep disturbance than fellow hostelers and that stress and sleep disturbances are important trigger factors for migraine.^11^, ^16^, ^19^ Comparing the results with that of study done on general population in Nepal also shows female preponderance, while the study has reported a higher prevalence (1‐year prevalence) than the findings of this study. This might be attributed to the altitude factors and easy accessibility of health facilities among medical students compared to general population.^15^ National level also study shows that odds of having migraine increases with increasing household altitudes but this could not be explored through this study as samples are taken from single city.^15^, ^20^

No significant difference was observed in year of study (preclinical or clinical) and migraine which is contrary to the previous studies; one Croatian study by Galinovic^21^ showed that more first‐year students visited clinics for migraine than final‐year students and another study by Ibrahim et al.^11^ reported higher migraine in second‐year students. This observation was likely due to increased stress among new medical students due to changes in the environment and strong academic demand for those coming from less stressful high‐school days. However, in our study, the COVID‐19 pandemic may have affected the finding, as the schedules were less hectic and the new coming medical students had ample time to adjust to the medical setting. The prevalence of TTH was found to be 40.3% in the current study within the global prevalence rate of 12%–78%.^22^ Studies from Syria,^23^ Turkey,^24^ and Nigeria^25^ reported much lower prevalence while another study from Saudi Arabia showed a similar prevalence.^26^ For TTH, no significant difference was with age, sex, year of study, and staying with the hostel, which is supported by findings of Alkarrash et al.,^23^ Syria.

This study is a nidus for future studies to what is yet to be studied in migraine and TTH among medical students. This study was done among the medical students studying at colleges of Kathmandu Valley only, while there are other colleges across the country where studies are yet to be conducted. This current study aims to establish the data of prevalence of migraine and TTH among undergraduate medical students for further big‐scale studies in the future, that can be of matter of interest to stakeholders who can intervene and introduce revisions in the medical education system for the sake of undergraduate medical students.

The major weakness of this study is that it has used a convenient sampling method and results might not be generalizable. Nonrandomized sampling method was used in this study (done among medical students) as this study is first of its kind in Nepal and the trend of probable outcome was not known. So, to obtain basic data quickly and with less complications convenient sampling was used. However, the basic data obtained from this study can act as nidus for future studies which will be more statistically rigorous and more generalizable.

## 5 CONCLUSION

In a nutshell, our study found the prevalence of migraine and TTH to be 15.3% and 40.3% respectively. There were higher odds of experiencing migraine headache with increasing age, higher odds in students who do not stay at hostel and females were at more odds than males. However, no such significant difference was observed in the case of TTH.

## AUTHOR CONTRIBUTIONS

Oshan Shrestha, Sagun Karki, Sunil Dhungel, and Dhan Bahadur Shrestha were involved in the conceptualization of the study. Oshan Shrestha, Sagun Karki, Niranjan Thapa, Kabindra Lal Shrestha, Aayushama Shah, Pramita Dhakal, and Prashant Pant were involved in data curation and initial manuscript drafting. Oshan Shrestha and Dhan Bahadur Shrestha did the formal analysis. Sunil Dhungel and Dhan Bahadur Shrestha edited the manuscript from an intellectual aspect. All the authors have read and approved the final version of the manuscript.

## CONFLICT OF INTEREST

The authors declare no conflict of interest.

## TRANSPARENCY STATEMENT

We affirm that the submitted manuscript is an honest, accurate, and transparent account of the study being reported; that no important aspects of the study have been omitted; and that any discrepancies from the study as planned (and, if relevant, registered) have been explained.

## ETHICS STATEMENT

Ethical clearance for this study was received from the Institutional Review Committee of the Nepalese Army Institute of Health Sciences (Ref no: 455). Consent to participate in the study was taken before the questionnaire page opened in Google form.

## Supporting information


**Supplementary File 1**: Sample size and Study tool.

**Supplementary File 2**: Univariate and Multivariate Logistic Regression results for TTH.

## Data Availability

Collected data that was analyzed is available from the corresponding author upon reasonable request.
