# Supplementary material for: Prevalence of migraine and tension‐type headache among undergraduate medical students of Kathmandu Valley: A cross‐sectional study
Source: Health Sci Rep. 2022 Aug 8;5(5):e747. doi: 10.1002/hsr2.747 (PMC9358540; doi:10.1002/hsr2.747)
Supplement: Supplementary file 1 — Sample size and Study tool. [file HSR2-5-e747-s001.docx]

**SAMPLE SIZE CALCULATION**

There are total of Six medical colleges that offer Bachelor of Medicine and Bachelor of Surgery (MBBS) program.

List of Colleges along with total students under MBBS program during the study period:

1. Maharajgunj Medical Campus: 404
2. Nepalese Army Institute of Health Science: 515
3. Patan Academy of Health Sciences: 325
4. Kist Medical College: 380
5. Kathmandu Medical College: 500
6. Nepal Medical College: 500

Total MBBS students at Kathmandu Valley: 2624

We used Cochran’s formula, considering the heterogenous and large population of medical students of Kathmandu Valley, for sample size calculation. Kathmandu is capital city of the country where students from all over the country pursue their study. Since the origin and the environment of the respective colleges under which they are studying makes the population under study heterogenous. Cochran’s formula is suited for population which is heterogenous and large.

n = Z ^2^ × (p × q) / e ^2^

= 1.96 ^2^ × (0.5 × 0.5) / 0.05 ^2^

= 384

where,

n = calculated sample size

Z = 1.96 at 95% Confidence Interval

p = expected prevalence of students having headache, 50%

q = 1-p

e = Margin of error (5%)

Total number of MBBS students in Kathmandu Valley during the study period (N): 2624

Adjusted sample size (n’) = n / (1+ n/N)

= 384/ (1+ 384/2624)

= 335

Considering 8% non-response rate, the final sample size was 361.

**STUDY TOOL**

**General Information**

Reference number:

Age:

Sex:

Year/Semester of study:

**General questions:**

I stay at college hostel: Yes/No (Y/N)

I am involved in extra-curricular activities: Y/N

I regularly exercise/meditate/do yoga/play outdoor sport: Y/N

I have a demanding family responsibility: Y/N

Food choice: Vegetarian/ Non-vegetarian

Smoking habit: Y/N

Co-morbidities: mention if any

Seek medical attention for your headache: Y/N

**HSQ- EV questionnaire:**

**Headache Screening Questionnaire – English Version**

1. How often in your life have you had a headache?

A. 1–4 times

B. 5–9 times

C. ≥ 10 times

2. Looking back at the last question, how often would you describe those headache moments as a headache-attack?

A. 0–4 times

B. 5–9 times

C. ≥ 10 times

3. How many days per month do you have headaches?

A. < 1 per month

B. ≥1 - <15 per month

C. ≥15 per month

4. How long does your headache last when you do not take any medication?

A. 0–30 min

B. 30 min–4 h

C. 4 h–3 days

D. 3–7 days

E. >7 days

5. What word would you use to describe your headache?

A. Pulsating feeling

B. Tight or pressing feeling

C. Burning or stabbing feeling

D. Other, such as

6. Is your headache one-sided or two-sided in nature?

A. One-sided

B. Two-sided

7. Describe the severity of your headache

A. Mild

B. Moderate

C. Severe

D. Very severe

**Indicate by the following statements if these are applicable to you when you have a headache.**

8. Daily activities (such as climbing stairs or walking) make my headache worse.

A. Yes

B. No

9. I avoid daily activities when I have a headache.

A. Yes

B. No

10. Describe what you experience during your headache (multiple answers possible).

A. Sensitivity to light

B. Sensitivity to sound

C. Nausea and/or vomiting

D. None of the above

E. Other, such as
