# Supplementary material for: Prevalence of migraine and tension‐type headache among undergraduate medical students of Kathmandu Valley: A cross‐sectional study
Source: Health Sci Rep. 2022 Aug 8;5(5):e747. doi: 10.1002/hsr2.747 (PMC9358540; doi:10.1002/hsr2.747)
Supplement: Supplementary file 2 — Univariate and Multivariate Logistic Regression results for TTH. [file HSR2-5-e747-s002.docx]

**Binomial Regression analysis for Tension-type Headache**

Univariate analysis showing crude odds ratio:

| **Variables** | **Coeff** | **S.E.** | **Wald** | **df** | **p** | **OR** | **95% C.I** | |
| --- | --- | --- | --- | --- | --- | --- | --- | --- |
|  |  |  |  |  |  |  | **Lower** | **Upper** |
| **Age** | -.056 | .068 | .671 | 1 | .413 | .946 | .827 | 1.081 |
| **Sex** | | | | | | | | |
| **Male** | Reference | | | | | | | |
| **Female** | .123 | .227 | .294 | 1 | .588 | 1.131 | .725 | 1.766 |
| **Year of Study** | | | | | | | | |
| **Pre-Clinical** | Reference | | | | | | | |
| **Clinical** | .004 | .221 | .000 | 1 | .984 | 1.004 | .651 | 1.549 |
| **Stays at hostel** | | | | | | | | |
| **Yes** | Reference | | | | | | | |
| **No** | -.074 | .230 | .104 | 1 | .747 | .928 | .592 | 1.457 |
| **Involved in Extracurricular activities** | | | | | | | | |
| **Yes** | Reference | | | | | | | |
| **No** | -.389 | .228 | 2.929 | 1 | .087 | .677 | .434 | 1.058 |
| **Daily exercise/yoga/outdoor sports** | | | | | | | | |
| **Yes** | Reference | | | | | | | |
| **No** | -.341 | .218 | 2.449 | 1 | .118 | .711 | .464 | 1.090 |
| **Have a demanding family responsibility** | | | | | | | | |
| **Yes** | Reference | | | | | | | |
| **No** | .096 | .232 | .172 | 1 | .678 | 1.101 | .699 | 1.735 |
| **Food preference** | | | | | | | | |
| **Vegetarian** | Reference | | | | | | | |
| **Non-Vegetarian** | .209 | .298 | .492 | 1 | .483 | 1.232 | .687 | 2.209 |
| **Smoking Habit** | | | | | | | | |
| **Yes** | Reference | | | | | | | |
| **No** | .548 | .412 | 1.771 | 1 | .183 | 1.729 | .772 | 3.875 |

Abbreviation: Coeff= Coefficient, S.E= Standard error, df= Degree of freedom, OR= Odds ratio, C.I= Confidence interval

Multivariate analysis showing adjusted odds ratio:

| **Variables** | **B** | **S.E.** | **Wald** | **df** | **p** | **AOR** | **95% C.I** | |
| --- | --- | --- | --- | --- | --- | --- | --- | --- |
|  |  |  |  |  |  |  | **Lower** | **Upper** |
| **Age** | -.051 | .087 | .353 | 1 | .553 | .950 | .802 | 1.125 |
| **Sex** | | | | | | | | |
| **Male** | Reference | | | | | | | |
| **Female** | .203 | .251 | .653 | 1 | .419 | 1.225 | .749 | 2.003 |
| **Year of Study** | | | | | | | | |
| **Pre-Clinical** | Reference | | | | | | | |
| **Clinical** | .083 | .273 | .092 | 1 | .761 | 1.086 | .636 | 1.855 |
| **Stays at hostel** | | | | | | | | |
| **Yes** | Reference | | | | | | | |
| **No** | -.043 | .238 | .032 | 1 | .858 | .958 | .601 | 1.528 |
| **Involved in Extracurricular activities** | | | | | | | | |
| **Yes** | Reference | | | | | | | |
| **No** | -.369 | .251 | 2.155 | 1 | .142 | .691 | .422 | 1.132 |
| **Daily exercise/yoga/outdoor sports** | | | | | | | | |
| **Yes** | Reference | | | | | | | |
| **No** | -.264 | .235 | 1.258 | 1 | .262 | .768 | .485 | 1.218 |
| **Have a demanding family responsibility** | | | | | | | | |
| **Yes** | Reference | | | | | | | |
| **No** | .080 | .240 | .111 | 1 | .740 | 1.083 | .677 | 1.734 |
| **Food preference** | | | | | | | | |
| **Vegetarian** | Reference | | | | | | | |
| **Non-Vegetarian** | .281 | .306 | .846 | 1 | .358 | 1.325 | .727 | 2.414 |
| **Smoking Habit** | | | | | | | | |
| **Yes** | Reference | | | | | | | |
| **No** | .551 | .429 | 1.655 | 1 | .198 | 1.736 | .749 | 4.020 |

Abbreviation: Coeff= Coefficient, S.E= Standard error, df= Degree of freedom, AOR= Adjusted odds ratio, C.I= Confidence interval
